# 3D Radiometric Mapping by Means of LiDAR SLAM and Thermal Camera Data Fusion

**DOI:** 10.3390/s22218512

**Published:** 2022-11-04

**Authors:** Davide De Pazzi, Marco Pertile, Sebastiano Chiodini

**Affiliations:** 1 School of Engineering, University of Padova, 35131 Padova, Italy; 2 Department of Industrial Engineering, University of Padova, 35131 Padova, Italy

**Keywords:** thermal camera, LiDAR, SLAM, data fusion, OcTree

## Abstract

The ability to produce 3D maps with infrared radiometric information is of great interest for many applications, such as rover navigation, industrial plant monitoring, and rescue robotics. In this paper, we present a system for large-scale thermal mapping based on IR thermal images and 3D LiDAR point cloud data fusion. The alignment between the point clouds and the thermal images is carried out using the extrinsic camera-to-LiDAR parameters, obtained by means of a dedicated calibration process. Rover’s trajectory, which is necessary for point cloud registration, is obtained by means of a LiDAR Simultaneous Localization and Mapping (SLAM) algorithm. Finally, the registered and merged thermal point clouds are represented through an OcTree data structure, where each voxel is associated with the average temperature of the 3D points contained within. Furthermore, the paper presents in detail the method for determining extrinsic parameters, which is based on the identification of a hot cardboard box. Both methods were validated in a laboratory environment and outdoors. It is shown that the developed system is capable of locating a thermal object with an accuracy of up to 9 cm in a 45 m map size with a voxelization of 14 cm.

## 1. Introduction

3D radiometric mapping is attracting growing attention in robotics, as it allows knowledge of the surrounding thermal scenario, making it possible to integrate mapping and path planning.

In this work, a 3D long-range radiometric mapping methodology aimed at indoor and outdoor environments has been developed. It was chosen to represent the surface temperature distribution map using an OcTree data structure. The advantages of this approach are numerous: (1) it allows the filtering and discretization of the thermal point cloud, (2) it allows us to volumetrically represent the thermal environment and for the robot to interact directly with it, and (3) compared to a point cloud, an OcTree data structure allows for a more computationally efficient representation of space. Compared to [1] we used an affordable solid-state LiDAR (LIVOX Horizon) characterized by a high detection range (up to 250 m). Furthermore, our method provides a direct thermal camera to LiDAR extrinsic calibration without going through the calibration with respect to an RGB camera. Furthermore, in this work, a metrological characterization of the mapping method was also carried out. The method uses the pose information obtained by reconstructing the rover’s trajectory with a LiDAR SLAM [2] algorithm as a basis for 3D point cloud registration. The association of the point-cloud elements with the radiometric information is obtained by performing the back projection of each LiDAR scan on the corresponding thermal image. The space is represented by an OcTree-based voxel grid, and for each occupied cell, the temperature obtained from the average of the temperatures of the points contained within is stored as the representative value.

The main contributions of this paper are as follows:

- Application of an extrinsic calibration method, originally developed to calibrate one or more optical cameras with respect to a 3D LiDAR, to the calibration of an IR camera system.
- Development of an accurate large-scale long-range thermal mapping method, capable of working even in the presence of poor lighting conditions in both indoor and outdoor scenarios.
- Development of an occupancy grid mapping based on thermal voxels stored in an OcTree object. The method offers the possibility of producing several maps at different resolutions (intended for different applications, e.g., navigation if low resolution and manipulation if high resolution) from the same data structure.
- Error analysis and study of the influence of the voxel size on the accuracy with which a thermal cluster is recognized.

The structure of the paper is divided as follows: in Section 2 is described the adopted extrinsic calibration technique, in Section 3 the three-dimensional thermal mapping method is presented, in Section 4 the experimental setup and preliminary results are shown, and in Section 5 the concluding remarks are reported.

## 2. Related Work

Thermography has a broad spectrum of applications ranging from rescue robotics to precision agriculture, industrial plant monitoring, and rover navigation. In the literature, many use cases can be found: Ulloa et al. [3] present a method for autonomous victim identification in a post-disaster environment based on thermal image processing and a computational neural network (CNN); Prashar et al. [4] highlight the benefit of infrared thermography as a tool for field phenotyping; Liu et al. [5] present a remote optical thermography detection method for silicone polymer insulating materials used in the power industry; Al-Habaibeh et al. [6] developed a method to evaluate thermal insulation performance of buildings using thermal imaging, while Cunningham et al. [7] present a slip prediction method for planetary rovers based on soil thermal inertial measurements.

Thermal mapping methods are mainly divided into four categories, as shown in Table 1. The point cloud that describes the geometry of the environment, which is going to be registered with thermal images, can be generated by means of: (1) Structure from Motion (SfM), (2) RGB-D sensor, (3) 3D laser and (4) 3D LiDAR SLAM. In the first category (thermal images registration over a point cloud generated by means of RGB images SfM) we can find the work of Ham and Golparvar-Fard [8]. The major drawbacks are the fact that this method leads to the generation of a sparse point cloud and fails under poor illumination conditions. Belonging to the second category (generation of 3D thermograms obtained by combining thermal images and RGB-D sensor) we can find the works of Muller et al. [9] and Vidas et al. [10,11]. Although they produce very detailed dense maps, these methods are limited to being used in indoor environments and to having a small operating range, due to the construction technology of RGB-D cameras. In the third category (registration method between infrared images and 3D laser scanner models), we can find the work of González-Aguilera et al. [12]. The system is capable of creating a very detailed model with long-range mapping capability for indoor and outdoor environments; however, the scan is limited to one position. Belonging to the fourth category are the methods based on LiDAR SLAM, the SLAM allows the registration of subsequent LiDAR scans, extending the operating range of this type of system. LiDAR SLAM is widely used for autonomous driving, precision agriculture, and industrial automation. A procedure for their characterization can be found in [13]. Oreifej et al. [1] presents a sensor suite comprising a set of 2D LiDARs, infrared cameras, and optical cameras, targeted at portable backpacks and capable of building 3D thermal maps. Borrmann et al. [14] proposes a system consisting of a mobile platform equipped with a 3D laser scanner, an RGB camera, and a thermal camera. Data from all sensors collected from various positions are combined into a common reference frame using calibration and scan matching using all detected data from all sensors together in the same reference frame.

## 3. Calibration Method

This section describes how to calibrate the thermal camera with respect to the LiDAR, to compute the extrinsic parameters $T_{Therm}^{LiDAR}=\left[R|t\right]$ required to perform a data fusion. The method used was originally developed and tested in [21] for the calibration of several RGB cameras with respect to a 3D LiDAR, and has been adapted to thermal cameras in this work. The adoption of this method was motivated by the need to repeat the calibration several times outside a laboratory environment, which would make it impractical to use a complex and cumbersome target. In addition, the need to use a target recognizable, both in the thermal images and in the point clouds, led to the discarding of checkerboard-like patterns (characterized by LiDAR scattering and plane fitting issues) in favor of a simpler three-dimensional geometry.

**Figure 1:**
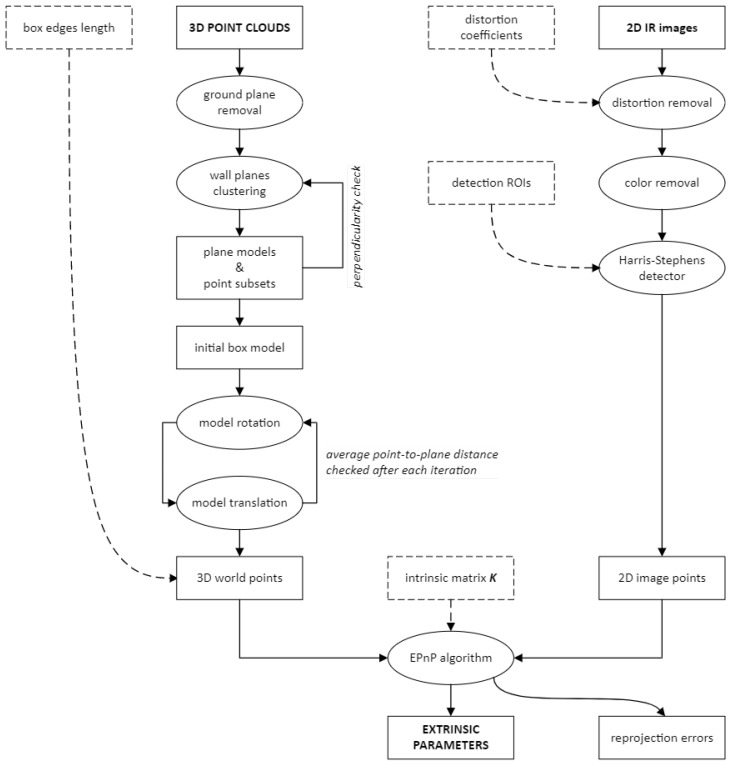
Block diagram of the camera-LiDAR calibration process. The algorithm takes a point cloud and a thermal image of the calibration target as input, returning the extrinsic parameters and the average reprojection error as output. The point cloud and the thermal image must have been acquired simultaneously, with the instruments in a fixed configuration. Each calibration dataset is processed separately, in order to select the one producing the lowest average reprojection error.

**Figure 2:**
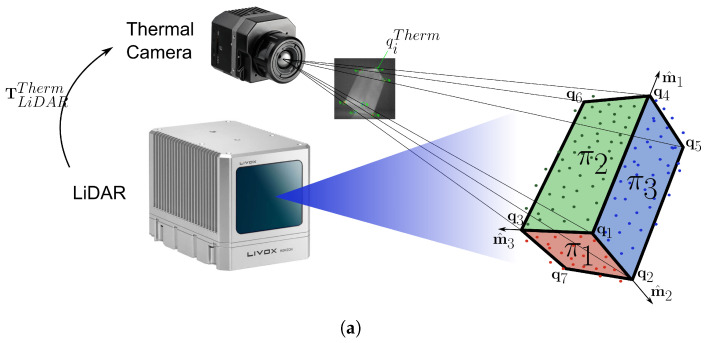

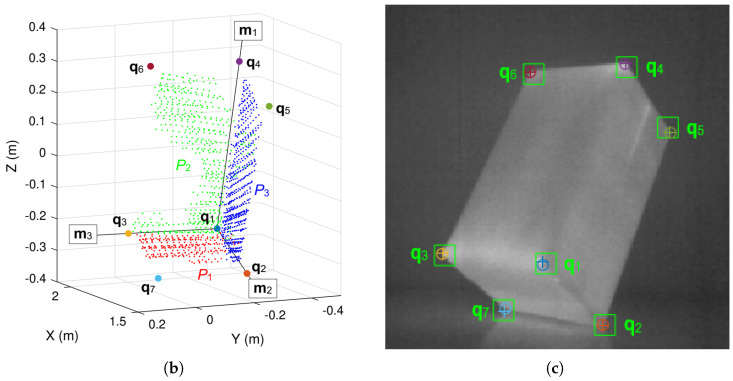
(**a**) Overview of the calibration process. (**b**) Refined target model produced by the calibration algorithm, showing the orientation of the edge vectors and the position of the corners. The line of sight of the instruments is parallel to the positive *X*-axis. Here, the box is seen from its right side. The front, side, and top walls (sorted by increasing elevation of the centroid) are colored red, blue, and green, respectively. If the box had been viewed from the left side, the reference frame would have been rotated (with the side wall in green and the top wall in blue) without changing the ordering of the corners. (**c**) Grayscale thermal image of the target, highlighting the regions in which the Harris-Stephens algorithm searches for each corner. Colored crosses mark the position of the image points and colored circles mark the position of the reprojected world points. Each point is represented with the same color as the corresponding corner in the 3D box model.

The calibration algorithm analyzes a 3D point cloud and a 2D thermal image of the same object to determine the pose of the thermal camera in the reference frame defined by LiDAR. Here, the calibration target is assumed to be an ordinary cardboard box of known size, which must be oriented so that it has three walls in full view of the instruments at the same time. The camera pose in the LiDAR reference frame can be found by solving a *Perspective-n-Point* (PnP) problem [22] that involves the seven visible corners of the box. The block diagram of the camera-LiDAR calibration process is shown in Figure 1, and a figure that gives an overview of the calibration process is shown in Figure 2a.

### 3.1. Point Cloud Processing

The first step of the proposed calibration process is to detect and cluster the walls of the box in the 3D point cloud. Each wall is represented by a subset $\pi_{i}$ of the original point cloud and by a parametric model $P_{i}=\left(A_{i},\;B_{i},\;C_{i},\;D_{i}\right)$ where

(1) $$A_{i}\;X+B_{i}\;Y+C_{i}\;Z+D_{i}=0$$

is the equation of a plane expressed in world coordinates (which coincide with the LiDAR’s reference frame) and

(2) $$n_{i}={\left\{A_{i},\;B_{i},\;C_{i}\right\}}^{T}$$

is the (non-unit) normal vector of that plane.

The ground plane can be detected and removed because, unlike any of the box walls, it can be assumed to be perpendicular to the vertical axis of the world frame. The algorithm then searches the groundless point cloud for the plane fit with the highest number of inliers, sets those points apart, and repeats the process to find two other planes. At this point, the remaining outliers are simply discarded.

The plane fitting function used here implemented a *M-estimator SAmple Consensus* (MSAC) algorithm [23] to identify the point cloud subset associated with each plane model. The planes found, however, will never be exactly perpendicular to each other (as the walls of the box actually are). Moreover, the inherent randomness of a RANSAC-based algorithm means that if no valid solutions are found, the process can be repeated to obtain a different result (as the calibration point clouds are expected to be of limited size, this step would not significantly increase the computational cost of the process). For this reason, the previous step must be repeated until the (unit) normals ${\hat{n}}_{i}$ of the three planes satisfy the following condition:

(3) $$|{\hat{n}}_{1}\cdot {\hat{n}}_{2}\left|+\right|{\hat{n}}_{1}\cdot {\hat{n}}_{3}\left|+\right|{\hat{n}}_{2}\cdot {\hat{n}}_{3}|\leq E_{max}$$

where $E_{max}$ is a user-defined threshold. Once a set of planes is accepted, the associated point cloud subsets $\pi_{i}$ are ordered according to the elevation of their centroids. The orientation of the target can then be inferred from the position of the second plane (the side face of the box) with respect to the other two (the front and top faces of the box).

### 3.2. Target Model Refinement

The second step of the calibration process is to create and refine a suitable model of the target, from which to obtain the three-dimensional coordinates of its visible corners.

The intersection of the three plane models $P_{i}$ returns three ordered edge vectors:

(4) $$\begin{array}{cc} \; & e_{1}={\hat{n}}_{2}\wedge {\hat{n}}_{3}\\ \; & e_{2}={\hat{n}}_{1}\wedge {\hat{n}}_{3}\\ \; & e_{3}={\hat{n}}_{1}\wedge {\hat{n}}_{2} \end{array}$$

and the single 3D point:

(5) $$q_{1}=-{\left[\begin{array}{ccc} A_{1} & B_{1} & C_{1}\\ A_{2} & B_{2} & C_{2}\\ A_{3} & B_{3} & C_{3} \end{array}\right]}^{-1}\left\{\begin{array}{c} D_{1}\\ D_{2}\\ D_{3} \end{array}\right\}$$

that is common to all of them. However, each edge vector can be thought of as the normal of one of the box walls, while $q_{1}$ corresponds to the corner closest to the instruments. Since the box walls and their normals are assumed to be perpendicular to each other, the box model (shown in Figure 2b) uses

(6) $$\begin{array}{cc} \; & m_{1}=e_{1}\\ \; & m_{2}=-e_{1}\wedge e_{2}\\ \; & m_{3}=m_{1}\wedge m_{2} \end{array}$$

in place of the original edge vectors. Each new vector points in the direction of the edge of the box it represents (that is, toward the “interior” of the box, as shown in Figure 2b) because its orientation is automatically adjusted by the algorithm.

This initial box model must be refined iteratively to better fit the original point cloud. Each iteration applies three rotations and three translations, in order to minimize the sum of squared distances of all points in each subset $\pi_{i}$ with respect to the corresponding plane model (identified by the point $q_{1}$ and the unit vector ${\hat{m}}_{i}$ parallel to $m_{i}$). When the whole model is rotated by an angle $\theta_{k}$ around each edge ${\hat{m}}_{k}$ (k=1,2,3) the cost function to be minimized is

(7) $$f_{k}\left(\theta \right)=\sum_{\begin{array}{c} i=1\\ i\neq k \end{array}}^{3}\sum_{j=1}^{N_{i}}{∥{\left(p_{j}^{i}-q_{1}\right)}^{T}R_{k}^{\theta }\;{\hat{m}}_{i}∥}^{2}$$

where $N_{i}$ is the number of points $p_{j}^{i}$ in the subset $\pi_{i}$ and $R_{k}^{\theta }$ is the axis-angle rotation matrix [24] for ${\hat{m}}_{k}$ and $\theta_{k}$. When the whole model has instead translated a distance $\delta_{k}$ along each edge ${\hat{m}}_{k}$ (k=1,2,3) the cost function to be minimized is

(8) $$g_{k}\left(\delta \right)=\sum_{j=1}^{N_{k}}{∥{\left(p_{j}^{k}-q_{1}-\delta \;{\hat{m}}_{k}\right)}^{T}{\hat{m}}_{k}∥}^{2}.$$

Here, the Levenberg-Marquardt algorithm [24] is used to solve the minimization problems. This algorithm (of standard use in non-linear optimization problems) was chosen because it is more robust than the Gauss-Newton algorithm: its slightly lower speed, on the other hand, is not an issue because the number of iterations required to optimize the box model is expected to be small. To update the model, each rotation is applied to the edge vectors and each translation is applied to the reference point. Iterations stop as soon as the average point-to-plane distance, computed over the whole model, falls below a given threshold.

Since the true length of the box edges can be measured, the three-dimensional coordinates of the unknown corners can be derived from the refined model as follows:

(9) $$\begin{array}{ccc} q_{2}=q_{1}+l_{2}\;{\hat{m}}_{2} & \; & q_{5}=q_{2}+l_{1}\;{\hat{m}}_{1}\\ q_{3}=q_{1}+l_{3}\;{\hat{m}}_{3} & \; & q_{6}=q_{3}+l_{2}\;{\hat{m}}_{2}\\ q_{4}=q_{1}+l_{1}\;{\hat{m}}_{1} & \; & q_{7}=q_{4}+l_{3}\;{\hat{m}}_{3} \end{array}$$

where $l_{k}$ refers to the edge identified by the *k*-th vector.

### 3.3. Thermal Image Processing

The intrinsic matrix of the thermal camera, together with the coefficients of radial and tangential distortion, was obtained with the Zhang method [25]. In this case, a flat checkerboard pattern of wood and aluminized tape, suitable for infrared detection, was used as the calibration target.

To obtain the two-dimensional image points corresponding to the three-dimensional world points identified in the previous step, an undistorted thermal image of the target is converted to grayscale and then processed with the Harris-Stephens algorithm [26] to extract the relevant features.

Each corner of the box is searched for inside a square area with a side of 15 pixels, whose position within the image (as shown in Figure 2c) must be defined manually before starting the calibration process. This is necessary to make sure that the image points are processed in the same order as the world points but also to prevent any unrelated feature of the target’s surface (or of the background) from being detected.

### 3.4. Extrinsic Parameters Determination

Knowing both the world coordinates and the corresponding image coordinates of all the visible corners of the box allows us to set up a PnP problem with n=7 points. The problem is then solved by an *Efficient PnP* (EPnP) algorithm [27]. This method addresses the general problem for n≥4 by expressing each of the *n* reference points as the weighted sum of four virtual non-coplanar control points, whose coordinates in the camera frame are treated as the unknown terms of the problem. Some implementations of the EPnP algorithm include a subsequent phase of iterative Gauss-Newton optimization to further refine the results. In any case, the solution can be found if at least four corners of the target have been successfully identified in both the point cloud and the thermal image.

The extrinsic parameters of the thermal camera with respect to the LiDAR consist of a rotation matrix R and a translation vector t such that

(10) $$\alpha \left\{\begin{array}{c} u\\ 1 \end{array}\right\}=K\left[R\;|\;t\right]\left\{\begin{array}{c} w\\ 1 \end{array}\right\}=P_{Therm}^{LiDAR}\left\{\begin{array}{c} w\\ 1 \end{array}\right\}$$

where $w={\left\{X,\;Y,\;Z\right\}}^{T}$ are the coordinates of a point in the world and $u={\left\{u,\;v\right\}}^{T}$ are the pixel coordinates of its reprojection on the image plane (for a given scale factor α). The intrinsic matrix of the thermal camera is

(11) $$K=\left[\begin{array}{ccccc} f_{u} & \; & s & \; & u_{c}\\ 0 & \; & f_{v} & \; & v_{c}\\ 0 & \; & 0 & \; & 1 \end{array}\right]$$

where $u_{c}$ and $v_{c}$ identify the optical center, $f_{u}$ and $f_{v}$ are the focal lengths in pixels and *s* is a skew coefficient. Here $P_{Therm}^{LiDAR}$ is a 3 × 4 world-to-image transformation matrix, so if the world point $q_{i}$ can be matched with a given image point $u_{i}$ then the resulting reprojection error can be computed as:

(12) $$\varepsilon_{i}=∥\left\{\begin{array}{c} u_{i}\\ 1 \end{array}\right\}-\frac{1}{\alpha }P_{Therm}^{LiDAR}\left\{\begin{array}{c} w_{i}\\ 1 \end{array}\right\}∥.$$

If multiple point clouds and thermal images are available, the calibration algorithm automatically selects the set of extrinsic parameters which leads to the lowest average reprojection error over all the *n* valid points.

## 4. Thermal Mapping Method

This section describes how a set of 3D LiDAR point clouds can be combined with a set of radiometric IR thermal images to create a three-dimensional representation of the temperature distribution in the environment the rover is traversing.

This process occurs in three steps: first, the color map and the temperature map extracted from each thermal image must be projected onto the corresponding point cloud, producing a sequence of thermal point clouds; the thermal point clouds are then registered and merged together into a single scene, using the pose information obtained by reconstructing the trajectory of the rover with a suitable LiDAR SLAM algorithm; finally, the registered and merged thermal point clouds are processed by a spatial decomposition algorithm to produce a voxel map, in which each cell of the 3D grid can be associated with a specific temperature value.

**Figure 3:**
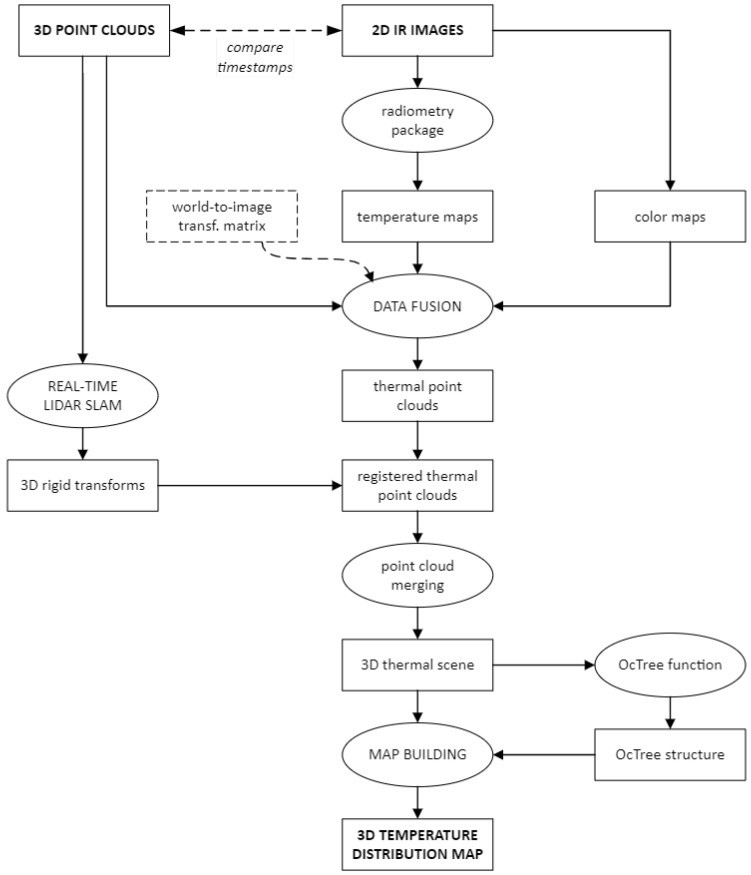
Block diagram of the mapping process. The algorithm takes a sequence of time-paired 3D point clouds and thermal images as input, returning a three-dimensional temperature distribution map of the scene as output. The 3D poses returned by the LiDAR SLAM (*livox_horizon_loam*) algorithm could also be used to create an occupancy map with the associated point clouds. Here the configuration of the camera-LiDAR system is expressed by the world-to-image transformation matrix $P_{Therm}^{LiDAR}=K\left[R\;|\;t\right]$ that was obtained through the extrinsic calibration process.

The map building is supposed to be performed offline, therefore all the point clouds produced by the LiDAR and all the radiometric images produced by the thermal camera are assumed to be available at the start of processing. The two datasets, however, must have been acquired at the same time, and with the instruments, in the same configuration they had when the extrinsic calibration was performed. Here the rover’s trajectory estimation is performed using the LiDAR SLAM algorithm *livox_horizon_loam* (https://github.com/Livox-SDK/livox_horizon_loam, accessed on 10 October 2022) (an improved version of LOAM *LiDAR Odometry And Mapping* algorithm [2]). This solution was chosen because it is a robust, relatively simple, and reasonably reliable method, with high drift compensation capabilities. The block diagram of the mapping process is shown in Figure 3.

### 4.1. Data Fusion

**Figure 4:**
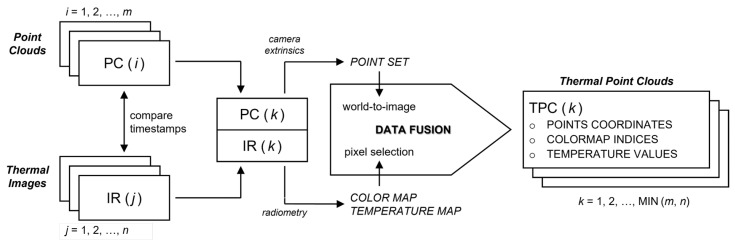
Overview of the data fusion procedure. The algorithm takes a sequence of 3D point clouds and thermal images as input, returning a set of 3D thermal point clouds as output. First, the timestamp of each point cloud PC(i) is compared with the timestamp of each thermal image IR(j) to extract a sequence $\left\{\mathrm{PC}(k),\;\mathrm{IR}(k)\right\}$ of time-paired couples. The data fusion itself consists in converting the world coordinates of each point in PC(i) to image coordinates, and using those coordinates to select a single pixel from the color map and the temperature map extracted from IR(k). This produces a sequence of thermal point clouds TPC(k) in which each element is characterized not only by its world coordinates but also by a color and a temperature value.

The data fusion between the LiDAR and the thermal camera consists of associating with each element of a point cloud the information from a specific pixel of the corresponding thermal image (that is, the values from a specific location in the color and temperature maps extracted from that image). An overview of the data fusion procedure is shown in Figure 4.

Equation (10) can be used to project the LiDAR points from the world coordinates to the image plane, as the camera has been calibrated with respect to the LiDAR and the projection matrix $P_{Therm}^{LiDAR}$ is known. Since the extrinsic parameters provided by the calibration algorithm minimize the reprojection errors, but doesn’t eliminate them, the data fusion function selects the pixel

(13) $$x_{i}=\left(r_{i},\;c_{i}\right)=\left(\left\lfloor v_{i}\right\rceil ,\;\left\lfloor u_{i}\right\rceil \right)$$

closest to the image point $u_{i}={\left\{u_{i},\;v_{i}\right\}}^{T}$ returned by (10) for a given world point. Both the color maps and the temperature maps extracted from the thermal images are matrices, so the vertical coordinate $v_{i}$ of the point determines the row index $r_{i}$ of the pixel and the horizontal coordinate $u_{i}$ of the point determines the column index $c_{i}$ of the pixel. As shown in (13), $u_{i}$ and $v_{i}$ are rounded to the nearest integer to identify the coordinates $c_{i}$ and $r_{i}$ of the pixel in question.

Temperature maps can be extracted from each image file using dedicated software provided by the manufacturer of the thermal camera. Each radiometric thermal image includes among its metadata the coefficients required to convert the pixel intensity values into the apparent temperature values. The coefficients are automatically computed by the internal radiometric software of the thermal camera, taking into account a number of parameters set by the user: air temperature, relative humidity, target emissivity, and approximate observation distance must be specified manually before any data can be collected. At present, the user-defined parameters can be set only once per image and are applied to all pixels, so they must necessarily represent average values compatible with most of the observed scene.

Before the thermal mapping process can begin, it is necessary to make sure that the point clouds and the thermal images acquired at the same time are correctly matched to each other. This means either associating each thermal image with a point cloud (if the dataset provided by the LiDAR has more elements than the one provided by the thermal camera) or associating each point cloud with a thermal image (if the dataset provided by the thermal camera has more elements than the one provided by the LiDAR). In both cases, the synchronization between the two datasets is performed by minimizing the absolute difference between their respective sets of timestamps.

After data fusion, all elements of each thermal point cloud carry two additional pieces of information: the color of a thermal image pixel (stored as an RGB triplet, to be used for the visual representation of the results) and the corresponding value from the associated temperature map (treated as a “point intensity” property in a standard point cloud model, for ease of processing). Note that 3D points outside the area included in the thermal camera’s FOV correspond to 2D points outside the image, and therefore their coordinates in the image plane will be either negative or greater than the size of the image itself. Such points are represented with the same out-of-palette color and given an invalid (i.e., NaN) temperature value, to be easily identifiable by both the user and the algorithm.

### 4.2. Point Cloud Registration and Merging

**Figure 5:**
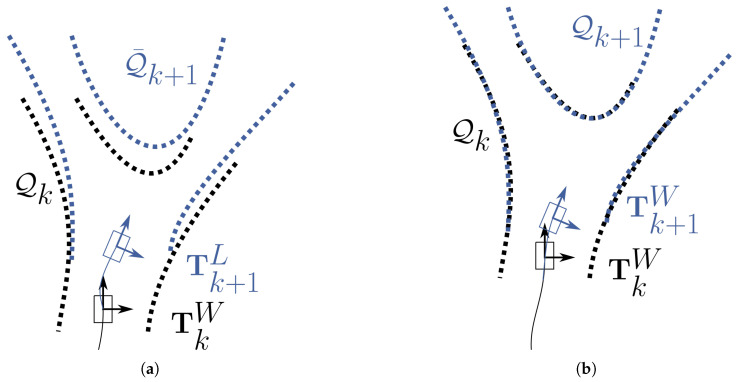

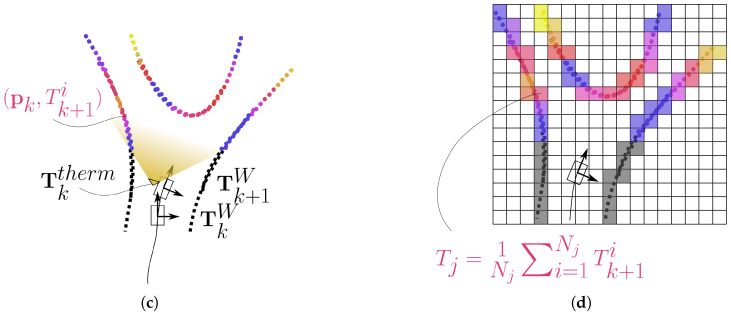
(**a**) Acquisition of the point clouds by the LiDAR while the rover is moving. Each set of points is initially expressed in its own reference frame. (**b**) Reconstruction of the trajectory by the LiDAR SLAM (LOAM) algorithm. The motion estimation $T_{k}^{L}$ provided by the odometry function is combined with the rover positioning $T_{k}^{W}$ determined by the mapping function to register the new point cloud ${\bar{Q}}_{k+1}$ (blue coloured) on the current map, returning the 3D transformation $T_{k+1}^{W}$ which adds a new segment to the reconstructed trajectory. (**c**) After the data fusion has been performed, the sequence of transformations $T_{1\cdots N}^{W}$ is used to align the thermal point clouds (rainbow coloured), before they are merged together. (**d**) The registered and merged thermal point clouds are then discretized with a 3D grid (here shown in section) by an *OcTree*-based spatial decomposition function. In the end, each cell is associated with the average temperature of the points it contains, producing a temperature distribution map.

The coordinates of the three-dimensional points acquired by the LiDAR are always expressed in the reference frame of the instrument, whose origin and orientation change continuously with respect to the environment as the rover moves (see Figure 5a): the thermal point clouds, therefore, must be registered before they can be merged into a single scene.

Registering a point cloud with respect to another, assuming that they overlap at least partially, means finding and applying the rigid transformation that minimizes an appropriate function of the distance between the two sets of points. If each point cloud can be aligned to the previous one, then, by composing all the related transformations, the entire sequence can be expressed in the reference frame of the first point cloud produced. Since the speed at which the LiDAR acquires the points is higher than the movement speed of the rover, motion-induced distortion in the point clouds is expected to be negligible. Moreover, it can be assumed that all transformations include only rotations and translations, without any scaling. In this case, the sequence of rigid transformations that allows one to register the thermal point clouds (see Figure 5c) is provided by the LiDAR SLAM algorithm (see Figure 5b) as the one which describes the rover’s trajectory.

The registered thermal point clouds can be merged together, preserving the shape and continuity of the structures (such as plane surfaces, edges, and corners) they contain. However, the merging cannot simply consist of grouping all the points into the same set: doing so would produce excessive concentrations of elements in the overlapping areas between two consecutive point clouds. Therefore, the algorithm processes the registered and merged thermal point clouds with a three-dimensional box filter. After the volume is divided into cubes of constant size, all elements within the same cube are replaced by a new point identified by averaging their coordinates. The purpose here is only to equalize the density of the resulting thermal scene, so the box filter can have a resolution (that is, a number of cells per unit length) much higher than the grid which will later be used for the spatial decomposition algorithm. Notice that the box filter affects all the properties of each point, including its temperature (which was stored as a “point intensity” value in the adopted point cloud model): this means that the new point which replaces all the point cloud elements within a given cubic volume will have a temperature value equal to the average of the temperatures of the point cloud elements it replaces.

Before proceeding with the spatial decomposition, the thermal scene must undergo some post-processing operations. First, to improve the efficiency of the spatial decomposition algorithm, the thermal scene is further downsampled by randomly selecting only a given fraction of its elements. Then the thermal scene could also be leveled by fitting a plane to the available points and determining the 3D rigid transformation that would make that plane normal to a certain reference vector, but only if the surface on which the rover is moving has a constant and known inclination. In all other cases, an “offset” described by the rotation matrix $R_{0}$ (computed for a roll angle ϕ and a pitch angle θ) could instead be applied. The two angles can be derived [28] using the classical relations

(14) $$\begin{array}{cc} \; & \phi =\mathrm{atan}2\left(a_{Y},\;a_{Z}\right)\\ \; & \theta =\mathrm{atan}2\left(a_{X},\;\sqrt{{a_{Y}}^{2}+{a_{Z}}^{2}}\right) \end{array}$$

where $a_{X}$, $a_{Y}$, and $a_{Z}$ are the linear accelerations provided by the LiDAR’s built-in IMU (or by other dedicated onboard sensors installed on the rover). Neither the downsampling nor the leveling affects the temperature values of the elements of the thermal scene.

### 4.3. Spatial Decomposition

In the previous steps of the thermal mapping process, a set of point clouds and a set of radiometric thermal images were combined into a thermal scene *S*

(15) $$S=\left(p_{k},\;RGB_{k},\;T_{k}\;|\;k=1,\cdots \;,N_{p}\right)$$

in which a 3D point $p_{k}={\left\{X_{k},\;Y_{k},\;Z_{k}\right\}}^{T}$ is also associated with a color $RGB_{k}$ and an apparent temperature $T_{k}$ (as shown in Figure 5c). Notice that the number of points $N_{p}$ can be very large, even for a relatively small scene.

The final step of the process aims to discretize the scene *S* with a three-dimensional grid, grouping its points into objects that represent finite portions of space. Each of these regions will be associated with the average temperature of the points it contains, building a 3D temperature distribution map with the same resolution as the grid used.

The algorithm used to decompose the scene is based on the *OcTree* model, a hierarchical data structure that recursively subdivides a volume into eight identical elements, also known as *voxels* [29]. This kind of decomposition can be represented by a “tree” whose branches could be pruned or merged to keep the structure memory efficient and quick to access. This form of decomposition was preferred over other possible solutions, such as those based on *kd-tree* data structures, because it guarantees a regular subdivision of space (that is, all the volume elements at a given level of an *OcTree* structure have the same shape and the same size).

To process the scene, the algorithm attaches to each voxel a *bin* of 3D points, represented by their coordinates and also by the index that identifies each of them in the original point cloud. The first bin encompasses the whole scene, and so contains all the points, but can be decomposed into eight children bins, which in turn could be decomposed further. Subdivision stops if an adequate condition is met, indicating that the required map resolution has been reached (or exceeded). Here, the stopping condition is expressed as a lower limit to the acceptable voxel edge length: the actual size of the voxels created, however, is determined only by the size of the volume to be decomposed and by the subdivisions required to reach that threshold.

In the end, to create a temperature distribution map, the bins (and the points they contain) must be processed back into *thermal* voxels (see Figure 5d): regions of space of known size characterized by a temperature value. Here, the algorithm selects only all the bins at a given level that contain at least 8 points, then recovers the temperature values of all the elements inside through their indices and averages them. The resulting voxel temperature

(16) $$T_{j}=\frac{1}{N_{j}}\sum_{i=1}^{N_{j}}T^{\;i}$$

can also be expressed as

(17) $$\tau_{j}=\left\lfloor 1+255\;\frac{T_{j}-T_{min}}{T_{max}-T_{min}}\right\rfloor$$

where $T_{max}$ and $T_{min}$ represent the maximum and minimum expected temperatures for the scene, respectively, while $\tau_{j}$ is used to select one of the 256 RGB triplets of a colormap. This allows us to represent the set of thermal voxels *V*:

(18) $$V=\left(w_{j},\;T_{j},\;\tau_{j}\;|\;j=1,\cdots \;,N_{v}\right)$$

as a set of colored cuboids, identified through the location $w_{j}$ of one of their corners (specifically, the closest to the origin of the world reference frame). Note that the number $N_{v}$ of voxels in *V* can be expected to be much lower than the number $N_{c}$ of points in *S*, as the resolution of the map should always be lower than the density of the thermal scene for the mapping process to be effective.

## 5. Experiments

**Figure 6:**
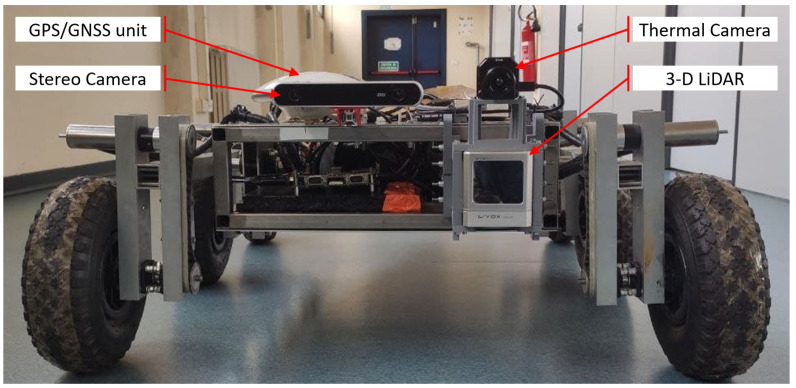
Instruments installed on board the MORPHEUS rover [30] when the proposed thermal mapping algorithm was tested outdoors. The 3D printed mounting structure, supporting the thermal camera and the LiDAR, was secured to the front of the rover’s frame (on the right), together with a Swift Navigation GPS/GNSS unit and a StereoLabs ZED binocular RGB stereo camera (on the left).

The experimental setup for testing the proposed calibration and thermal mapping algorithms consists of a FLIR Vue Pro R 336 radiometric thermal camera and a Livox Horizon 3D LiDAR. One of the notable features of the LiDAR used is the adoption of a non-repeating scanning pattern, which gradually increases FOV coverage over time (as shown in Table 2). The two sensors can be seen in Figure 6.

The thermal camera and the LiDAR were placed on a 3D-printed mounting structure and then calibrated. The calibration algorithm described in Section 3 analyzes only specific portions of the input thermal image, and the input point cloud can be cropped so as to include only the target box and its immediate surroundings. This allowed us to acquire the calibration datasets in an environment that was not specifically modified for such a purpose: the only precaution adopted was to briefly heat the target with a heat gun, to produce a suitable thermal contrast with respect to the (much colder) background.

### 5.1. Indoor Test

**Figure 7:**
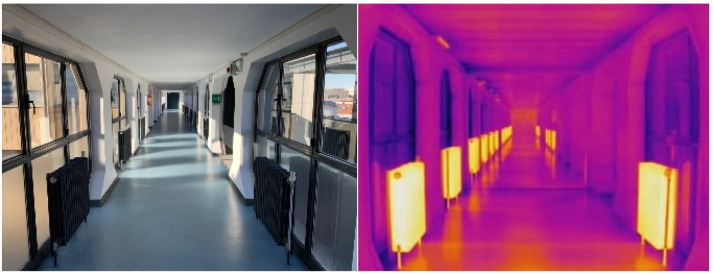
Visual (**left**) and thermal (**right**) images of the indoor environment used to test the proposed algorithm. Notice how the thermally distinctive elements on both sides of the corridor are still recognizable in the thermal scene (Figure 8) as well as in the corresponding temperature maps (Figure 9a,b).

**Figure 8:**
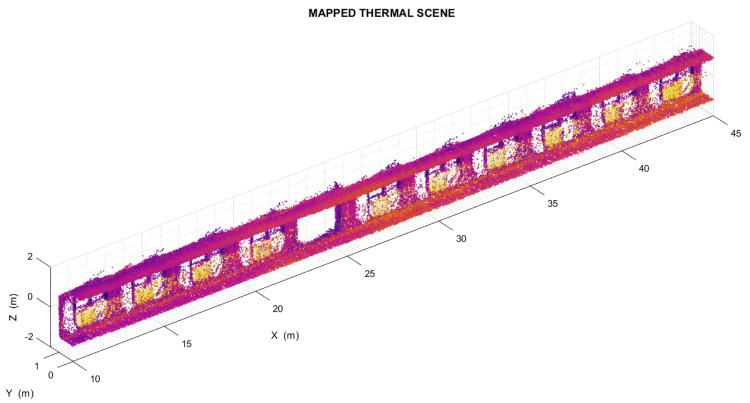
Left side of the thermal scene produced during the test in the indoor environment.

To test the proposed thermal mapping method in an indoor environment, the instrument mounting (together with a laptop computer and the external battery used to power the LiDAR) was temporarily placed on a small cart and pushed down a flat straight corridor (shown in Figure 7). This area contains a number of thermally distinctive elements, which are regularly arranged throughout its length. These elements can be clearly recognized in the thermal scene (Figure 8) and therefore should appear on the corresponding 3D temperature distribution map as easily identifiable voxel arrangements.

**Figure 9:**
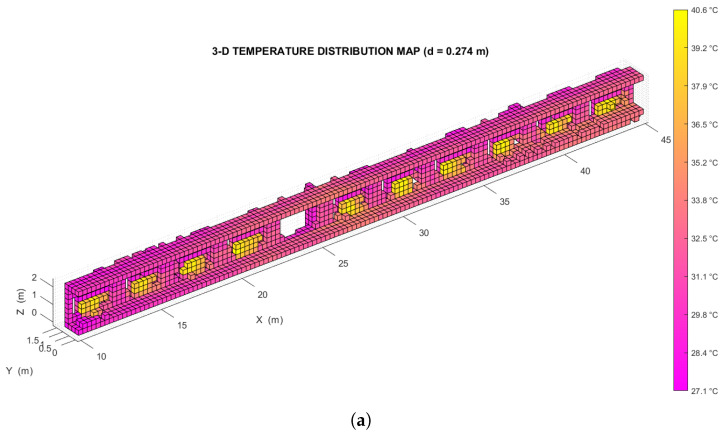

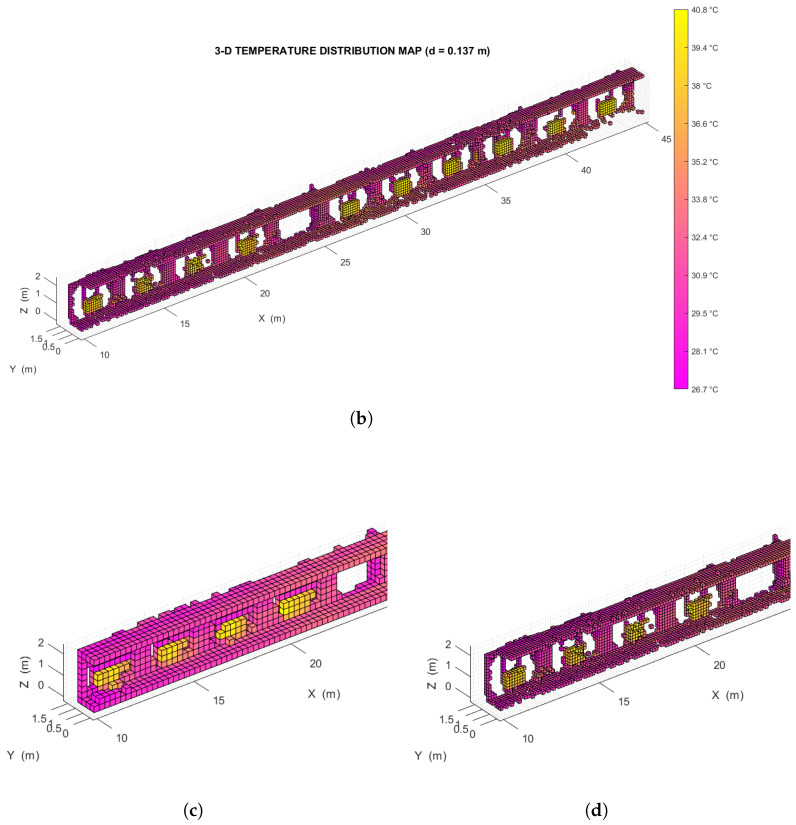
(**a**) Left side of the low resolution temperature distribution map relative to the indoor test environment. (**b**) Left side of the high resolution temperature distribution map relative to the same environment. (**c**) Detail of the low resolution indoor temperature distribution map. (**d**) Detail of the high resolution indoor temperature distribution map.

The analysis was performed on two temperature distribution maps of the same environment, each of which was divided into two halves to simplify the representation of the results:

- A *low resolution* map, with a voxel edge size of 0.27 m resulting in ∼3.7 voxels/m (shown in Figure 9a).
- A *high resolution* map, with a voxel edge size of 0.14 m resulting in ∼7.1 voxels/m (shown in Figure 9b).

The two maps were created from the same OcTree data structure, so they describe the exact same set of points. Specifically, the high resolution map represents the subdivision from the ninth level of the source structure, while the low resolution map represents the subdivision from the eighth level of the same structure.

**Figure 10:**
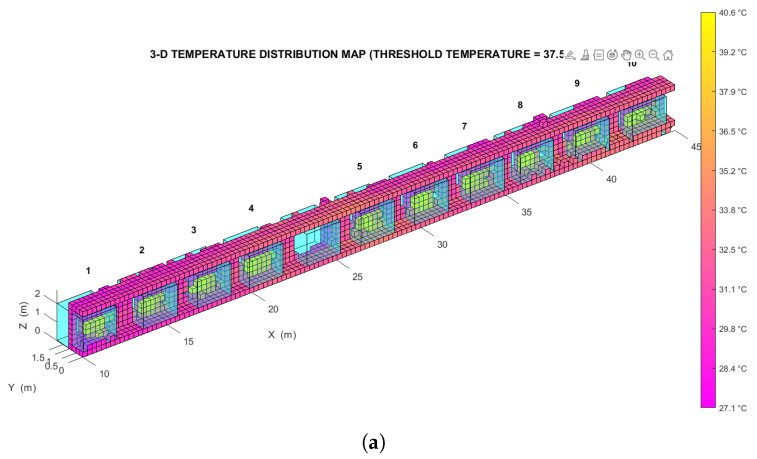

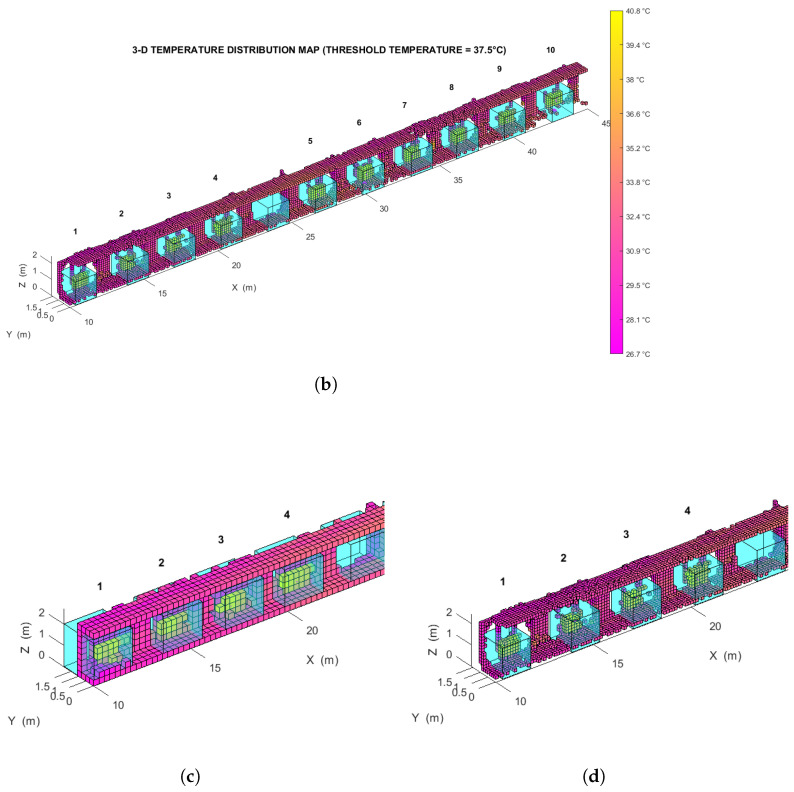
(**a**) Voxel clusters detected, with a threshold temperature of $37.5{\;}^{\circ }$C, on the left side of the low resolution temperature distribution map relative to the indoor test environment. (**b**) Voxel clusters detected, again with a threshold temperature of $37.5{\;}^{\circ }$C, on the left side of the high resolution temperature distribution map relative to the indoor test environment. (**c**) Detail of the clusters in the low resolution indoor temperature distribution map. (**d**) Detail of the clusters in the high resolution indoor temperature distribution map.

To determine whether this map accurately describes the test environment, the spatial distribution of its thermal voxels was analyzed automatically: all voxels with a temperature above a suitable threshold (in this case, $T_{th}=37.5{\;}^{\circ }$C) were extracted and grouped into separate *clusters*, each made up of at least 3 voxels in close proximity to each other (as shown in Figure 10a,b). The distance between these clusters, whose location could be found by averaging the coordinates of the centroids of all their constituent voxels, was compared with the distance between the environmental features that they represent. The spacing between the thermally distinctive elements was previously measured on-site, using a measuring tape whose accuracy of 1 mm is approximately an order of magnitude greater than that achieved by the proposed method.

**Figure 11:**
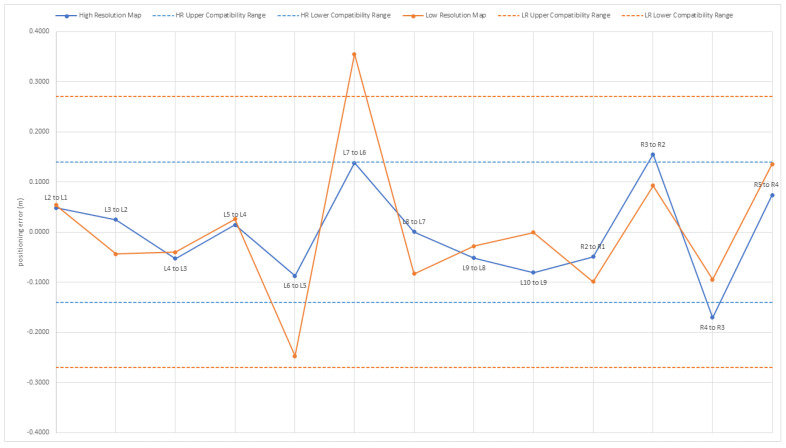
Distance errors relative to the temperature distribution maps produced in the indoor test environment. Dashed lines delimit the compatibility range in the two cases, which is equal to plus or minus the size of one voxel. The error standard deviation has been computed for both the low resolution map and the high resolution map.

The plot in Figure 11 illustrates the results of this analysis in the two cases considered, showing the distance error (that is, the difference between the estimated and the expected separation along the X−axis) for each pair of consecutive clusters, on the left and right side of the corridor. Note that to account for the discretization of the thermal scene, a maximum difference equal to the size of a voxel is still considered acceptable. The standard deviation of the error for the low resolution map is 0.145 m, and the standard deviation of the error for the high resolution map is 0.093 m.

These metrological performances are slightly better than those obtained in the previous research work of [14] where the positioning error obtained is equal to 4 cm. Considering that its test was carried out in a room with a maximum size of 5 m, it leads to a relative error of 1%. We obtain a positioning error of 9 cm (by making a 14 cm voxelization) in an environment with a maximum length of 45 m, which corresponds to a relative error of 0.2%.

### 5.2. Outdoor Test

**Figure 12:**
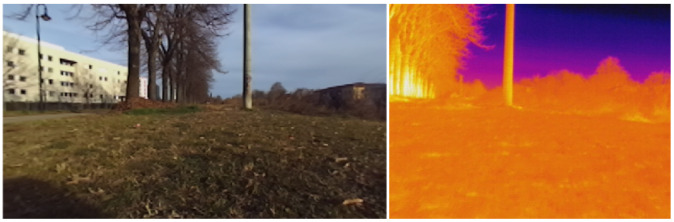
Visual (**left**) and thermal (**right**) images of the outdoor environment used to test the proposed algorithm.

**Figure 13:**
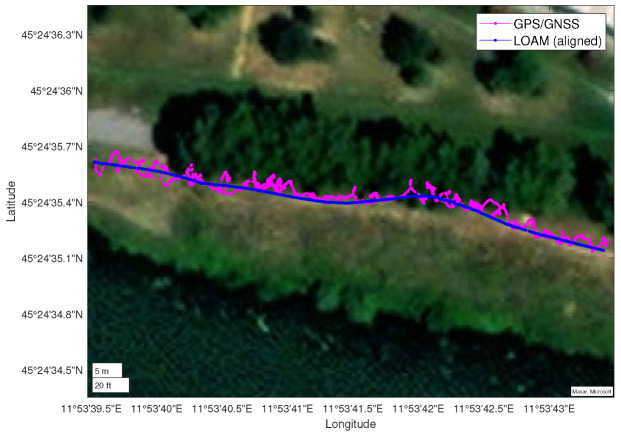
Comparison between the trajectory reconstructed in real-time using the *livox_horizon_loam* algorithm (blue) and the path traced by the rover’s GPS/GNSS receiver (purple). Here the Horn’s quaternion-based algorithm was used to align the two paths and to represent the reconstructed trajectory in the same latitude/longitude/altitude coordinate system used for the GPS/GNSS points.

To test the proposed thermal mapping method in an outdoor environment (shown in Figure 12), the mounting with the thermal camera and the 3D LiDAR was installed on the MORPHEUS rover [30] (shown in Figure 6), designed and built by the students of the University of Padova [30]. When the rover is moving outdoors, the output of the SLAM algorithm can be compared with the positioning data provided by an onboard GPS/GNSS receiver. In this case, Horn’s quaternion-based method [31] was used to align the two paths (as shown in Figure 13) and evaluate their compatibility.

**Figure 14:**
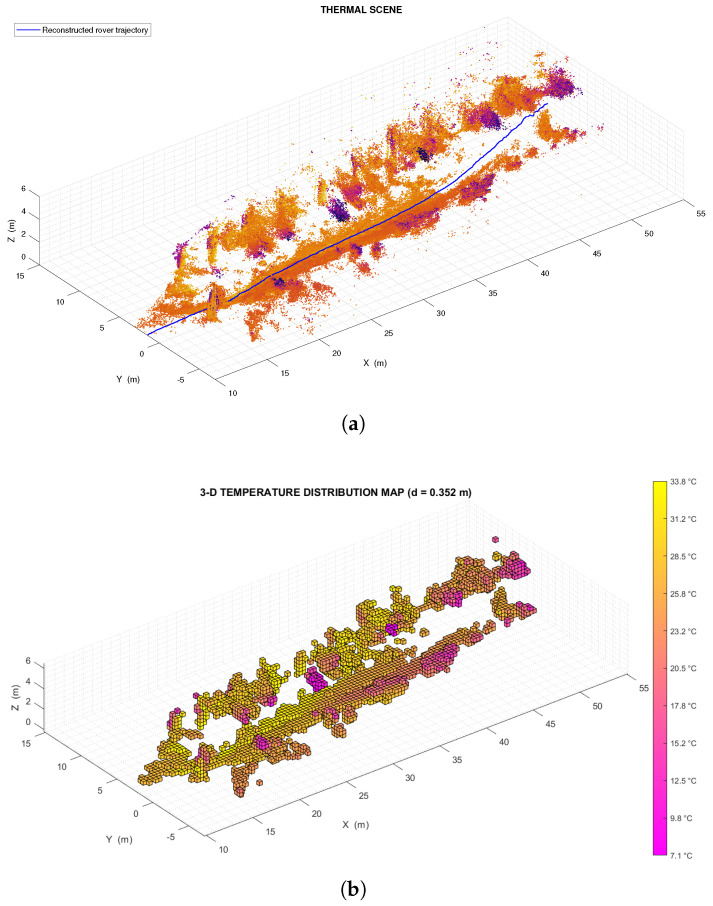

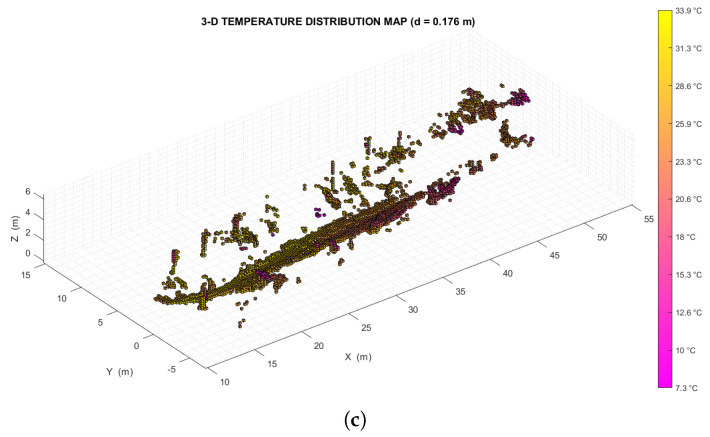
(**a**) Portion of the thermal scene produced during the test in the outdoor environment. Here the blue line represents the estimated trajectory of the rover, which was reconstructed using the *livox_horizon_loam* algorithm. (**b**) Low resolution (voxel size equal to 0.35 m) temperature distribution map relative to the outdoor test environment. (**c**) High resolution (voxel size equal to 0.18 m) temperature distribution map relative to the outdoor test environment. Again, the high resolution map was obtained from the same OcTree data structure as the low resolution temperature distribution map (with the difference being that the first map corresponds to the eighth level of the structure, and the second to the ninth level).

This second test was intended primarily to demonstrate that the *livox_horizon_loam* algorithm is capable of reconstructing the trajectory of the rover (as shown in Figure 13 and Figure 14a), thus providing a sufficiently valid basis for the registration of the thermal point clouds, and that the thermal mapping algorithm is capable of producing temperature distribution maps of large size (handling a large number of points and voxels).

## 6. Conclusions

This article describes a thermal mapping method based on data fusion between a 3D LiDAR and a thermal imaging camera. The target application sees thermal environment monitoring via UGVs (*Unmanned Ground Vehicles*). The calibration method used to align the reference systems of the LiDAR and the thermal imaging camera is also presented. LiDAR point cloud registration with the UGV relative pose reconstruction was based on a LiDAR SLAM algorithm (*livox_horizon_loam*). For efficient representation of the 3D temperature distribution map, a discretization with a voxel grid by means of an OcTree-based spatial decomposition function was performed. The method was characterized in an indoor environment by tracing the presence of hot elements, such as radiators. The standard deviation of the localization error of a thermally significant element has been shown to be equal to 0.145 m for a low resolution map (voxel size equal to 0.27 m) and a standard deviation of 0.093 m for a high resolution map (voxel size equal to 0.14 m). The two maps were created from different levels of the same OcTree structure, that is from the same set of thermally-characterized 3D points. The method was also tested outdoors, by integrating the sensors onboard a UGV. The tests performed showed how adopting too low a map resolution reduces the complexity of the representation, but also its level of detail (which could prevent the detection of some of the features within the map itself). On the other hand, adopting too high a map resolution leads to the number of voxels being comparable to the number of points, preventing the temperature values from being averaged significantly.

## Figures and Tables

**Table 1:** Existing 3D radiometric mapping methods.

Approach	Advantages	Disadvantages
Structure from Motion (SfM) [8,15,16]	• Long operating range	• Generates sparse map
	• Outdoor and indoor	• Fails with poor illumination
	• Lightweight and affordable	• No absolute scale information
RGB-D [9,10,11,17]	• Generate dense map	• Small operating range
	• Lightweight and affordable	• Limited to indoor environments
3D-laser [12,18,19]	• Long-range mapping capability	• Bulky and expensive
	• Outdoor and indoor	• Scan limited to one location
	• Accurate	
3D-LiDAR SLAM ([1,14,20] and proposed work)	• Long-range mapping capability	• Large amount of data
	• Long operating range	
	• Accurate	
	• Lightweight and affordable	
	• Generate dense map	

**Table 2:** Instrument specifications.

Thermal Camera	
Detector Technology	Uncooled VOx Microbolometers
Spectral Region	7.5–13.5 μm (LWIR)
Detector Resolution	336×256 pixels
FOV	$35^{\circ }\times 27^{\circ }$ (9 mm optics)
**3D LiDAR**	
	90 m @ reflectivity 10%
Maximum Detection Range	130 m @ reflectivity 20%
	260 m @ reflectivity 80%
Minimum Detection Range	0.5 m
FOV	$81.7^{\;\circ }\times 25.1^{\circ }$
FOV Coverage	60% @ 0.1 s
	98% @ 0.5 s
Point Acquisition Rate	>240,000 points/s
Random Error (1−σ @ 20 m)	0.02 m (distances)
	$0.05^{\circ }$ (angles)

## Data Availability

Not applicable.

## Abbreviations

The following abbreviations are used in this manuscript:

CNN	Computational Neural Network
EPnP	Efficient Perspective-*n*-Point
FOV	Field Of View
GNSS	Global Navigation Satellite System
GPS	Global Positioning System
IMU	Inertial Measurement Unit
IR	InfraRed
LiDAR	Light Detection And Ranging
LOAM	LiDAR Odometry And Mapping
LWIR	Long-Wave InfraRed
MSAC	M-estimator Sample Consensus
NaN	Not a Number
PC	Point Cloud
PnP	Perspective-*n*-point
RANSAC	RANdom SAmple Consensus
SfM	Structure from Motion
SLAM	Simultaneous Localization And Mapping
TPC	Thermal Point Cloud
UGV	Unmanned Ground Vehicle
VOx	Vanadium Oxide
